# Effectiveness of Mindfulness and Positive Strengthening mHealth Interventions for the Promotion of Subjective Emotional Wellbeing and Management of Self-Efficacy for Chronic Cardiac Diseases

**DOI:** 10.3390/jpm12121953

**Published:** 2022-11-25

**Authors:** Carmen Tabernero, Tamara Gutiérrez-Domingo, Patrizia Steca, Rosario Castillo-Mayén, Esther Cuadrado, Sebastián J. Rubio, Naima Z. Farhane-Medina, Bárbara Luque

**Affiliations:** 1 Instituto de Neurociencias de Castilla y León (INCYL), University of Salamanca, 37007 Salamanca, Spain; 2 Department of Social Psychology, University of Salamanca, 37005 Salamanca, Spain; 3 Maimonides Biomedical Research Institute of Cordoba (IMIBIC), 14004 Cordoba, Spain; 4 Department of Psychology, University of Cordoba, 14071 Cordoba, Spain; 5 Department of Psychology, University of Milan-Bicocca, 20126 Milan, Italy; 6 Department of Specific Didactics, University of Cordoba, 14071 Cordoba, Spain

**Keywords:** cardiovascular disease, intervention, positive and negative wellbeing, mindfulness, positive strengthening, cardiovascular management self-efficacy

## Abstract

Intervention in health prevention and treatment via mobile phones is becoming a key element on health promotion. Testing the efficacy of these mobile health (mHealth) psychological interventions for cardiovascular health is necessary as it is a chronic pathology, and it can influence the affective and cognitive states of patients. This research aimed to analyze the effectiveness of two brief psychological interventions—mindfulness and positive strengthening—to promote subjective emotional wellbeing and disease management self-efficacy using mHealth. This was a three-arm intervention and feasibility study, with a pre-post design and three follow-up measures with 105 patients (93 completed all phases) with cardiovascular diseases. Group 1 and 2 received the mindfulness or strengthening intervention, and Group 3 was the control group. The positive–negative affect and management self-efficacy for chronic and cardiovascular diseases were analyzed over time, while anxiety and depression levels were assessed at the beginning of the study. The results showed that mindfulness and positive strengthening interventions both had a positive effect on participants’ affective state and management self-efficacy for the disease in comparison with the control group over time, even after controlling for baseline anxiety and depression levels. Positive strengthening seems to be more effective for improving cardiac self-efficacy, while mindfulness practice was significantly more effective at reducing negative affect at the first face-to-face evaluation.

## 1. Introduction

Cardiovascular diseases (CVDs) are the leading cause of morbidity and mortality worldwide [1]. Consequently, improving the wellbeing and cardiovascular health (CVH) of patients is one of the priorities of preventive guidelines in cardiology [2]. The occurrence of psychological comorbidities related to emotional and psychological distress is frequently associated with risk factors for CVDs [3]. Thus, different theoretical models, such as the stress buffering model [4,5], propose that interventions to increase positive affect and subjective emotional wellbeing in patients with CVDs should be a priority in order to help them out of this negative affective spiral.

Research on the effectiveness of psychological interventions in CVH suggests the positive impact of mindfulness interventions [6] (learning to focus attention on the present moment with full awareness) and positive strengthening interventions [7,8] (based on positive psychology they focus on cultivating positive psychological constructions through systematic exercises) to increase the wellbeing of adults with CVDs. Participants undergoing these interventions experienced a greater reduction of anxiety, depression, and perceived stress [8,9], a reduction in their blood pressure [10], and an improvement in physical and psychological wellbeing [6,11].

Another relevant psychological construct for CVH is self-efficacy [12], according to which a high perception of the ability to cope and persevere in the face of difficulties is an effective self-regulatory mechanism to improve physical and mental health. In this sense, CVDs may affect the patient’s quality of life and perception of their ability to exert control over their own functioning and the demands of challenges, such as disease management and rehabilitation [13]. Research has shown evidence of a positive influence of perceived self-efficacy for CVD management on the motivation to follow a healthy diet and the patients’ quality of life [14].

Given that one of the main sources to generate self-efficacy in individuals is through experiencing emotional states [12], psychological interventions focused on improving the subjective emotional wellbeing of patients with CVDs could facilitate a greater perception of self-efficacy for managing their chronic condition. Considering the fact that chronic disease management requires sustained care over time, mobile health (mHealth) interventions appear as a suitable approach when devising psychological care for patients with CVDs. The recent COVID-19 pandemic situation has also fostered mHealth interventions. Moreover, mHealth interventions are proving to be a novel and effective alternative to promote healthy behaviors in patients with CVDs from a self-management approach [15], and a recent meta-analysis has shown promising results related to the effectiveness of home-based cardiac telerehabilitation [16]. Thus, the need to intervene in the psychological effects associated with CVDs [17] requires interventions in this health condition to be tested and adapted to the new technological developments. This technology is also more cost-effective and therefore leads to a greater financial savings for healthcare systems. In view of the above, we developed a feasibility study that could help to further explore this issue and expand research on the use of technology for psychological interventions in patients with CVDs. Specifically, this study aimed to test the efficacy of two brief mHealth psychological interventions on the increase in patients’ self-efficacy judgments to manage their chronic cardiac disease and their subjective emotional wellbeing.

### 1.1. Subjective Emotional Wellbeing and Management Self-Efficacy for Cardiovascular Health

The study of subjective emotional wellbeing [18], which involves considering affective evaluations with the predominance of positive over negative affect, has become a very important topic in the field of health [19]. So too is the hedonic view, whereby attributes such as pleasurable experiences, happiness, positive affect, and life satisfaction influence psychological wellbeing and disease progression [20]. In a longitudinal study of patients who had suffered a myocardial infarction, Kroemeke [21] analyzed changes in subjective emotional wellbeing and found that affective balance was modified from a prevalence of negative affect in the initial phase to a more positive affect over time. Physiological states experienced in circumstances characterized by high anxiety, stress, and fatigue influence mood and personal confidence regarding the ability to overcome such circumstances [12]. Therefore, the physiological states that accompany the affective states can influence self-efficacy, either positively or negatively, and vice versa [12]. There is evidence that self-efficacy for the management of chronic diseases [22] and, specifically, CVDs [23] influences affective balance and health perception. Moreover, a cardiac rehabilitation intervention on the management of emotions has shown a positive impact on the management of the disease in cardiac patients [24].

Mobile health interventions (mHealth), that is, interventions implemented through mobile devices with the aim to improve health, could be presented as a novel and effective alternative to promote healthy behaviors in cardiac patients through a self-management approach [16]. Miller [25] has reported that health prevention and treatment intervention through mobile phones is becoming a key element in health research. One of the most widely used instant messaging service is currently WhatsApp, which may have a positive impact on the engagement of patients. Thus, Burke et al. [26] have reviewed the evidence for the use of mHealth in the prevention of CVDs, concluding that, despite existing limitations, mHealth has shown potential to modify lifestyles by promoting changes in health behavior. Similarly, Legler et al. [27] have conducted a study on the impact of positive psychological intervention based on the post-acute high-risk coronary syndrome period via mobile messaging to assess whether cardiac patients can improve their wellbeing and promote health behaviors associated with greater survival. Likewise, testing the efficacy of these mHealth psychological interventions to promote cardiovascular health is a challenge for future research.

### 1.2. Mindfulness Interventions

Research suggests that self-management of health can be improved through mindfulness via three pathways: attention control, emotion regulation, and self-awareness [28]. Several studies have been conducted suggesting that a mindfulness-based intervention can be an effective strategy to address mental health and wellness conditions in different populations [29,30]. Mindfulness programs have been conducted and shown to be effective, such as a brief psychological training intervention to reduce depressive symptoms, [31] and to improve subjective wellbeing in cardiac patients [32]. Likewise, Kraft et al. [33] conducted a mobile message-based intervention to reduce depressive symptoms through mindfulness practice, and an online mindfulness-based program has been used to study the efficacy of this strategy to improve affect and its positive impact on anxiety and depression using the recording of physiological measurements [34].

### 1.3. Positive Strengthening Interventions

Positive psychology interventions, which use brief activities focused on the promotion of happiness through an increase in gratitude, positive affect, optimism, and personal strengths, are related constructions that have been widely studied [7]. Specifically, in the field of CVH, positivity has been effective in improving patient wellbeing after acute coronary syndrome [35]. Likewise, Sanjuán et al. [36] performed a program based on positive interventions in cardiac patients, achieving improvement in their emotional state, and Nikrahan et al. [37] have found that these interventions can help improve wellbeing by reducing the biomarkers of cardiovascular risk. Furthermore, the benefits of gratitude—one of the most used strategies in positive interventions—on different measures of wellbeing, in which positive and negative affect are measured, has been explored by Rash et al. [38], who concluded that grateful contemplation could be used to improve long-term wellbeing. In this sense, the physiological correlates of gratitude have been studied which may explain the positive effects on subjective wellbeing and physiological health [39] and inflammatory markers [40].

### 1.4. Objectives and Hypotheses

The main aim of this three-arm intervention and feasibility study was to longitudinally test the effectiveness of two brief mHealth psychological interventions over time in patients with CVDs. Therefore, the main hypotheses were that both psychological interventions—mindfulness and positive strengthening—H1a) will improve subjective emotional wellbeing (increasing positive affect and reducing negative affect) and H2a) will increase the personal beliefs about management self-efficacy (chronic disease and CVDs) of cardiac patients compared to a ‘treatment as usual’ (TAU) group. Moreover, these effects were expected to be maintained at follow-up measurements (two and four weeks after the end of the intervention) for both emotional wellbeing and self-efficacy (H1b and H2b, respectively).

## 2. Materials and Methods

### 2.1. Participants and Procedure

The sample was obtained from the cardiology unit of the Reina Sofía University Hospital of Córdoba (HURS), Spain. The study was approved by the Research Ethics Committee of the Servicio Andaluz de Salud (Act 242, Ref. 2886, 29 June 2015). Participation was completely voluntary; participants were informed of the research objectives before giving their consent to participate and did not receive any reward for their participation.

In total, 179 patients were invited to participate. The inclusion criteria were having a diagnosis of a CVD and being older than 18 years old. The exclusion criteria were having a cognitive impairment (e.g., dementia), sensory impairment (e.g., visual impairment), or severe mental disorder (e.g., schizophrenia); in this regard, 26 patients were excluded because they reported any of the aforementioned difficulties. In addition, 39 patients declined to participate. This allowed us to have greater control of possible extraneous variables and to avoid problems with message tracking and understanding. In addition, 9 patients did not attend the appointment. Thus, the total number of study participants was 105 patients (*Mean age* = 64.2 years, *SD* = 10.8 years) with CVDs (mainly angina pectoris, myocardial infarction, arrhythmia, or heart failure). The study results are based on the final sample of 93 patients who completed all of the phases of the study (*Mean age* = 63.92 years, *SD* = 10.92). A priori power analysis with G*power software [41] was conducted, using an F-test as the test family and “ANOVA with repeated measures, for a within-between interaction” as the statistical test, entering 3 as the number of groups, 4 as the number of measurements, and 0.3 as the correlation among repeated measures (assuming a somewhat low correlation among repeated measure because some variability could be expected across time in the study variables, particularly in the experimental groups). An alpha value of 0.05 and a power of 0.80 were assumed as is generally carried out [42]. In addition, a Cohen’s f of 0.25 was requested, as this value is the lower end for a medium effect size, thus allowing it to be conservative [43]. Finally, calculations were made considering both that the sphericity assumption was met and that it was not met. The epsilon value for non-sphericity correction was 0.5, as this is the most stringent for three conditions [44]. This analysis yielded an optimal total sample size of 42, considering that the sphericity assumption is met (actual power 0.82), and an optimal total sample size of 66, considering that the sphericity assumption is not met (actual power 0.82). Therefore, the study sample seemed to be sufficient for the main analysis performed with a medium effect size.

**Figure 1:**
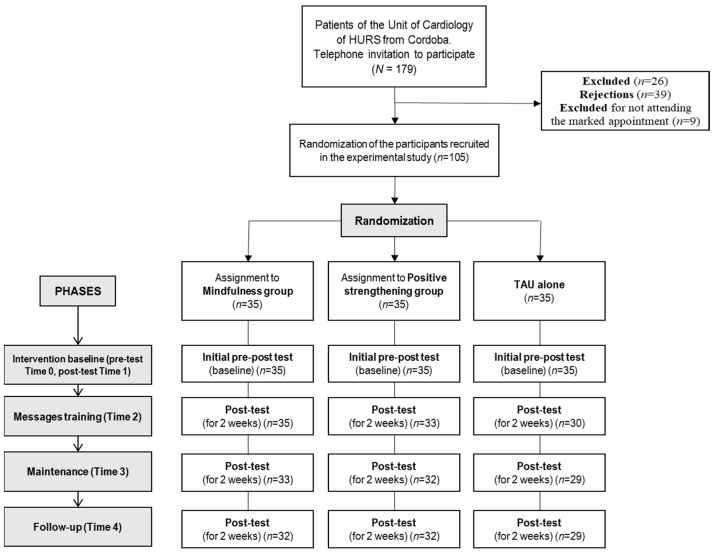
CONSORT (Consolidated Standards of Reporting Trials) diagram. Recruitment and study performance. HURS = Reina Sofía University Hospital.

This was an intervention and feasibility study, with a three-arm (two experimental groups and one TAU group) and pre-post design, comparing two types of brief psychological interventions. First, cardiac patients from the cardiology unit of the HURS were contacted by telephone. The recruited patients available to attend the face-to-face session were randomly assigned to one of the experimental conditions: the mindfulness intervention (N = 35) or the positive strengthening intervention (N = 35). Participants unavailable to attend this session were the TAU group (N = 35). As recommended in the TREND statement for reporting the participant flow [45], Figure 1 shows the flow of participants at each stage of the study by using the CONSORT diagram [46].

Participants in the intervention conditions were called for the in-person intervention session. These sessions were always taken by the same researcher, a qualified psychologist with experience in mindfulness and positive psychology interventions. It included an initial presentation about the study, signing of the informed consent form, the pre-test questionnaire (baseline), a brief specific psychological intervention that exemplifies the psychological training strategy, and then the first post face-to-face session (post-session). Subsequently, for two weeks, a text message was sent to their mobile device every day, through WhatsApp, with the activity they had to perform. Once the training was over, the post-test questionnaire was returned to them (post-test 1). Consequently, they carried out a maintained activity for another two weeks, after which the post-test evaluative measure was repeated (post-test 2). Finally, after two weeks of follow-up, the last post-test evaluation was carried out (post-test 3). Each survey was carried out through the Unipark Questback online platform, and each participant completed a total of three post-tests by phone.

### 2.2. Interventions

#### 2.2.1. In-Person Intervention

Prior to the mHealth intervention, participants from both experimental groups attended to a face-to-face session, which was designed to be equivalent in structure, sample size, amount of instruction, and practical activity at home for each intervention approach. Each session lasted approximately one hour, and the content included information on the type of specific intervention (what it consists of, its practical use, and its benefits), real-life training on-site, and recommendations to be followed for the practical training activity at home.

#### 2.2.2. mHealth Intervention

The psychological training via the mobile application involved for both approaches, mindfulness and positive strengthening, daily practice of a specific activity instructed via text message to the participant’s mobile device through WhatsApp, with one message every day for two weeks. The online messages were always sent at the same time, ensuring that they were brief and understandable by using accessible language (see Supplemental File S1). The TAU group continued with the conventional monitoring suggested by the cardiologist.

1. Mindfulness mHealth intervention. The mindfulness intervention consisted of formal and informal mindfulness-based exercises (e.g., breathing awareness, body scan, exploration through the senses). We adapted a mindfulness-based stress reduction program [47] for instruction by messaging with the specific objective of full attention every day (see Supplemental File S1). The practice based on breathing awareness was carried out for 10 min. The goal was to develop awareness in cardiac patients through this approach based on contact with the present moment and full attention.
2. Positive strengthening mHealth intervention. The program of activities was adapted by our research team from Seligman’s approach [7] (see Supplemental File S1). For this intervention, activities were proposed for a positive assessment of the main vital areas of the participant’s life (personal, family, social, and daily life). The objective of the program was to reinforce the importance of focusing on the good things that happen to us each day, combined with gratitude exercises and assessment of personal achievements from the perspective of positive psychology.

After completing the training administered by mobile messaging through WhatsApp, a period of activity was maintained for another two weeks to prolong the daily practice. The participants in the mindfulness group followed a simple plan that would allow them to be more focused on the present and instructed them to reserve 10 min each day to connect with themselves through deep breathing. The participants from the positive strengthening group were instructed to reflect on three good things each night before going to sleep as a positive moment of their day.

#### 2.2.3. TAU Group

Participants in this group did not receive any specific psychological intervention but continued their usual medical follow-up (periodic revisions, cardiologist appointments, analytical monitoring, etc.).

### 2.3. Measures

#### 2.3.1. Positive and Negative Affect

The Positive and Negative Affect Schedule [48], which evaluates mood through positive and negative affect, was used to evaluate the level of subjective wellbeing of the participants, alongside positivity. The instrument consists of 20 items and uses a Likert scale of five points, where 1 = “totally disagree” and 5 = “strongly agree”: 10 items (e.g., “excited”) measure positive affect and 10 items (e.g., “worried”) measure negative affect. The Cronbach’s alpha coefficients in the original study were 0.87 and 0.86, respectively; in a Spanish sample of patients with CVDs, the reliabilities of the measure were 0.88 and 0.80, respectively [49]. In the current sample, the reliability for positive affect was α = 0.89 at baseline and α = 0.91 at post-test 3, and for negative affect, the reliability was α = 0.87 at baseline and α = 0.89 at post-test 3.

#### 2.3.2. Cardiovascular Management Self-Efficacy Scale (CMSES)

The CMSES [23] was used to evaluate the management self-efficacy beliefs of CVD patients. The participants responded to nine items designed to evaluate three main dimensions, namely cardiac risk (e.g., “How well you can avoid problems or difficult situations and reduce sources of stress”), adherence to therapy (e.g., “Remember to take daily medication, even when there is nobody to remind you about it”) and recognition of symptoms (e.g., “How well you recognize the signs of worsening of your illness and understand when you need to call your doctor”), on a Likert scale of five points, where 1 = “not at all confident” and 5 = “completely confident”. The Cronbach’s alpha coefficient in the original study was 0.68; in our sample, it was α = 0.77 at baseline and α = 0.69 at post-test 3.

#### 2.3.3. Self-Efficacy for Managing Chronic Disease Scale (SEMCD)

The SEMCD [22] was used to measure self-efficacy changes in evaluations of chronic disease self-management. Participants responded to six items (e.g., “How confident are you that you can keep the emotional distress caused by your disease from interfering totally with the things you want to do?”) on a Likert scale of 10 points, where 1 = “not at all confident” and 10 = “totally confident”. In the original study with cardiac patients, the Cronbach’s alpha coefficient was 0.88, and in the current sample, it was α = 0.88 at baseline and α = 0.91 at post-test 3.

#### 2.3.4. Anxiety and Depression

The Hospital Anxiety and Depression Scale (HADS) [50] uses two factors to assess the symptoms of anxiety and depression. For this scale, participants responded to 14 items divided into two factors (e.g., “I feel tense or nervous” as an anxiety item and “I feel as if I am slowed down” as a depression item) on a Likert scale of four points with a range of responses 0–3. In the original study, the Cronbach’s alpha coefficients were 0.80 and 0.81 for anxiety and depression, respectively; in a Spanish sample of patients with CVDs the Cronbach’s alpha coefficients were 0.86 and 0.80, respectively [51], and in the current sample, they were 0.80 and 0.84 at baseline.

#### 2.3.5. Engagement and Satisfaction

Personal assessment of the brief psychological interventions was evaluated with two questions: (a) the degree of engagement of the patients with each task (i.e., the frequency with which they completed it) was evaluated objectively for each of the follow-up timepoints (1 = “never”, 2 = “sometimes”, 3 = “moderately”, 4 = “almost always”, and 5 = “every day”), and (b) participants’ general satisfaction (from 1 to 10) with the program to improve their wellbeing.

### 2.4. Data Analyses

First, regarding the demographic characteristics, the means and standard deviations between the groups were evaluated, and the baseline differences between the groups were analyzed using the χ^2^ test for discrete variables and analyses of variance (ANOVA) for continuous variables. As preliminary results, one-way ANOVA and multivariate ANOVA were conducted to explore the effects of the face-to-face session and test any differences between the experimental groups. Subsequently, a 3 × 4 repeated-measures ANCOVA for each study variable (positive affect, negative affect, cardiovascular self-efficacy, and chronic disease self-efficacy) was carried out using the type of intervention with three levels as the between-subject variable (*experimental condition*: mindfulness group, positivity group, and TAU group), the phases of measures with four levels as the within-subject variable (*time*: baseline, post-intervention, follow-up 1, and follow-up 2), with anxiety and depression as covariates. These analyses were carried out to evaluate differences between pre- and post-evaluations. When Mauchly’s test of sphericity was significant, degrees of freedom were corrected by Greenhouse–Geisser’s correction or by Huynh–Feldt’s correction if Greenhouse–Geisser’s ε was >0.75 [42]. Pairwise comparisons and post-hoc analyses were adjusted by Bonferroni’s correction multiple tests, and partial η^2^ was calculated to estimate the magnitude of intervention effect sizes. Rules of thumb based on Cohen’s F [52] were used for interpretation purposes, considering η_p_^2^ 0.01, 0.06, and 0.14 as small, medium, and large effects, respectively. All statistical analyses were carried out using SPSS software (26.0). Considering the exploratory nature of this feasibility study, complete-case analyses were performed.

## 3. Results

The demographic characteristics of the sample for each group are described in Table 1. The results did not show differences between the groups in the main variables: age, gender, marital and employment status, education and economic level, and time living with and type of CVD. On the other hand, when we compared the age at the onset of disease, there were significant differences between groups. Specifically, post-hoc analysis through the Bonferroni parameter showed significant differences between the mindfulness and positive strengthening groups (t = 6.97, *p* < 0.05), and between the TAU and positive strengthening groups (t = 8.91, *p* < 0.01).

As preliminary results, the potential changes produced in the study variables after the face-to-face intervention were explored. Repeated measures ANOVA were carried out considering the two intervention groups (group: mindfulness and positive strengthening) at baseline and after the face-to-face session (time: baseline, post-session). The factor time was significant for each variable, with higher positive affect, *F*(1,62) = 44.21, self-efficacy for managing the chronic disease, *F*(1,62) = 22.85, and cardiovascular management self-efficacy, *F*(1,62) = 15.85, and lower negative affect, *F*(1,62) = 96.17, after the in-person session, all *p*s < 0.001. However, the interaction effect group x time was not significant, all *F*s < 1.97, all *p*s > 0.165. Pairwise comparisons adjusted by Bonferroni’s correction for multiple tests only showed a significant difference in negative affect, with the mindfulness group showing lower negative affect than the positivity group (mean difference = −0.25, *p* < 0.04).

A 3 × 4 repeated measures ANCOVA was conducted for each dependent variable, with anxiety and depression as covariates, to test the effect of the psychological interventions at the different study phases. For a better readability of the results, Table 2 shows the within-subject effects.

For positive affect, Mauchly’s test indicated that the assumption of sphericity had been violated, χ^2^(5) = 22.61, *p* < 0.001, therefore degrees of freedom were corrected using Huynh–Feldt estimates of sphericity (ε = 0.95). The results indicated a significant main effect of time and of the interaction between time x experimental condition. Concerning negative affect, Mauchly’s test indicated that the assumption of sphericity had been violated, χ^2^(5) = 32.15, *p* < 0.001, therefore degrees of freedom were corrected using Huynh–Feldt estimates of sphericity (ε = 0.90). The test of within-subject effects showed a main effect of time and significant interaction effects of time x anxiety and time x experimental condition.

As for cardiovascular management self-efficacy, given that Mauchly’s test was significant, χ^2^(5) = 52.52, *p* < 0.001, a correction of the degrees of freedom was made using Greenhouse–Geisser estimates of sphericity (ε = 0.70). The results showed a significant main effect of time and significant interaction effects of time x depression and time x experimental condition. Regarding self-efficacy for managing chronic disease, given that Mauchly’s test was significant, χ^2^(5) = 11.61, *p* = 0.041, a correction of the degrees of freedom was made using Huynh–Feldt estimates of sphericity (ε = 0.99). A significant main effect of time was observed, as well as for the interaction effects of time x depression and time x experimental condition.

**Figure 2:**
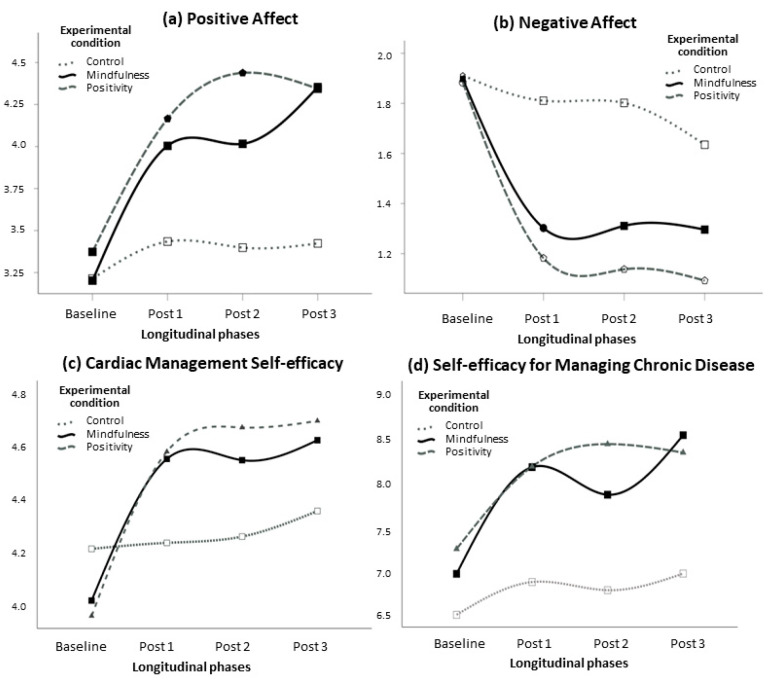
Longitudinal effects of experimental interventions (mindfulness and positive strengthening) and control on positive and negative affect, cardiac management self-efficacy, and self-efficacy for managing chronic diseases (depression and anxiety were inserted as covariates with values of 3.47 and 3.14, respectively).

The graphics presented below (Figure 2) show the marginal estimated means for both intervention groups (mindfulness and positive strengthening) versus the TAU group at baseline and at the three post-measurement times: after two weeks training (Post 1), after two weeks maintenance (Post 2), and after two weeks follow-up (Post 3), for positive (Figure 2a) and negative affect (Figure 2b), cardiac management self-efficacy, (Figure 2c) and self-efficacy for managing chronic disease (Figure 2d).

Pairwise comparisons adjusted by Bonferroni’s correction showed a significant difference between the TAU group and the mindfulness group (*M*_difference_ = −0.49, *p* < 0.001) and the positivity group (*M*_difference_ = −0.71) for positive affect; the same pattern was found for negative affect (*M*_difference_ = 0.38, *p* < 0.001 and *M*_difference_ = 0.58, *p* < 0.001, respectively), and for self-efficacy for managing the chronic disease (*M*_difference_ = −0.96, *p* = 0.001 and *M*_difference_ = −1.20, *p* < 0.001, respectively). As for cardiac management self-efficacy, a significant difference was found between the TAU and the positivity groups (*M*_difference_ = −0.17, *p* = 0.039). Concerning the effect of the interventions over time, all pairwise comparisons adjusted by Bonferroni’s correction indicated significant differences between baseline and measurements at post-intervention, follow-up 1, and follow-up 2 for positive affect, negative affect, cardiac management self-efficacy, and chronic disease management self-efficacy, all *p*s < 0.001. For positive affect, a significant difference was also found between post-intervention and follow-up 2 measurements. Overall, the results showed that, once the interventions were implemented and after controlling for anxiety and depression levels, participants in both experimental groups reported higher levels of positive affect, CMSE and SEMCD and lower levels of negative affect than the participants of the TAU group. These differences were maintained at the two follow-up measurements.

Results related to personal assessment of brief psychological interventions showed no significant differences between the intervention groups in engagement and general satisfaction at any of the post-test evaluations (Table 3).

## 4. Discussion

The aim of this intervention and feasibility study was to test the effectiveness of two brief psychological interventions delivered via mHealth technology on subjective emotional wellbeing and self-efficacy to manage the disease in patients with CVDs. Overall, the results suggested that the two psychological interventions had a positive effect after completion of the intervention via WhatsApp and after two- and four-week follow-up.

The preliminary results indicated that the face-to-face mindfulness intervention had a greater positive impact than the positive strengthening intervention on cardiac patients, resulting in a lower negative affect. The link between mindfulness and a reduction in negative emotions has been studied previously by Fogarty et al. [53]. Similarly, Kroemeke [21], in a longitudinal study of myocardial infarction patients, observed that the affective balance was modified to a more positive affect over time, with problem-focused coping strategies mediating positive affect changes. Regarding the relationship between cognitive and affective variables, some authors [54] have associated the mindfulness-based stress-reduction program with the development of problem-focused coping strategies.

To test the study hypothesis, given prior evidence on the relationship between CVDs and the affective symptoms of anxiety and depression in the patients [3], and as carried out in previous research [55], these two psychological variables were used as covariates in each analysis in order to test the effects of the psychological interventions on subjective emotional wellbeing (increasing positive affect and reducing negative affect) and management of cardiac and chronic disease self-efficacy over time. As expected, the results revealed that the interaction of the study phases and the experimental groups was significant for all psychological variables analyzed (positive and negative affect, cardiac management self-efficacy, and self-efficacy for managing chronic disease). These results suggest that both psychological interventions had an impact on the scores of the study variables, varying over time and across the different experimental conditions, even after controlling for anxiety and depression. Specifically, concerning subjective emotional wellbeing, the patients with CVDs that participated in any of the psychological interventions (mindfulness or positive strengthening) showed higher scores on positive affect and lower scores on negative affect than participants of the TAU group at the post-intervention phase, that is, after completing the mHealth intervention (Hypothesis 1a). The participants of the experimental groups also reported higher scores in cardiovascular management self-efficacy and self-efficacy for managing chronic disease than participants of the TAU group, suggesting a positive influence of the mHealth intervention on the personal beliefs on management self-efficacy in patients with CVDs (Hypothesis 2a). However, pairwise comparisons revealed that, for cardiac self-efficacy, there was a significant difference between the positivity group and the TAU group, but the scores of the last group did not differ from the scores of the mindfulness group. This different effect of the psychological interventions, together with the findings concerning the face-to-face session, suggests that positive strengthening might be preferred for improving self-efficacy beliefs, while mindfulness practice would be more appropriate for improving emotional wellbeing or, more specifically, at least for reducing negative affect. Although emotional state is taken into account in both interventions, this difference seems reasonable if attention is paid to the specific contents of each intervention approach: the mindfulness intervention’s focus on awareness and full attention might facilitate improvements in emotional wellbeing, whereas the combination of gratitude exercises and the re-evaluation of personal achievements might allow for improvements in cardiac self-efficacy, especially in patients with CVDs.

In relation to our predictions of the effects of the psychological interventions over time, the results analyzing the follow-up measurements indicated that both the mindfulness and positive strengthening programs were shown to have a significant influence on subjective emotional wellbeing (increasing positive affect and reducing negative affect, hypothesis 1b), and confidence regarding self-efficacy for managing cardiac and chronic disease (hypothesis 2b) over time compared with the TAU group. Importantly, the effect size of the interaction between the study phases and the experimental condition was medium except for self-efficacy for managing chronic disease. This different effect size of the self-efficacy measures could be explained because of the higher specificity of the cardiac management self-efficacy scale for patients with CVDs than the self-efficacy for managing a chronic disease measure.

In our study, two specific self-efficacy beliefs were evaluated: cardiac management self-efficacy—focused on the management of cardiac risk, adherence to therapy and recognition of symptoms [23]; and self-efficacy for managing chronic disease—focused on the self-management of emotional distress caused by the disease [22]. As described above, our results showed that both brief interventions enhanced the management self-efficacy for chronic and cardiac disease, particularly mindfulness practice. This result is concordant with those reported by Marogna et al. [24], who found that, after a cardiac rehabilitation intervention, the management of emotions had a positive impact on self-management of the disease. It is important to note that the two interventions tested in this study focused on emotional factors and, therefore, the results regarding the improvement of self-efficacy for managing chronic disease are a sign of the effectiveness of both interventions in providing patients with greater confidence in their ability to handle the emotional distress caused by the disease; moreover, neither of the interventions was directly focused on the development of behavioral and cognitive abilities for managing cardiac disease, adherence to therapy, or recognition of symptoms, which supports the connection between emotional wellbeing and self-efficacy development.

Finally, our results did not show significant differences in the frequency and general level of satisfaction between the two brief psychological interventions scored at the three post-test times. This could be because both interventions followed a similar design pattern (WhatsApp). The use of mHealth for health promotion interventions is a promising development [15,25,56], and our study can shed new light in this direction.

### 4.1. Strengths

Although there were some dropouts in each of the intervention groups and in the control group, participants of the experimental groups were generally highly committed over time. This may be related to the type of format used, based on mHealth, which allowed the participants to be monitored remotely, thus facilitating their adherence to the study. In fact, the increasing wave of mHealth interventions [25] has promised favorable results in health promotion. Nonetheless, more mHealth studies with innovative designs are required to collect further evidence [57], and the results of this study might help to add more data to this topic.

The intervention was carried out through mHealth due to its simplicity, which meant minimal burden for both participants and researchers compared to other types of more complex and more time-consuming interventions, such as mobile applications. Likewise, it allowed free access to the participants, without any structural barriers. Patients were able to find a practical, simple activity in the messages that adapts adequately to their daily routine and lifestyle. In light of the results obtained, we consider the text messages to have followed the recommended parameters for mHealth [57]. With WhatsApp being one of the most used instant messaging services currently, it seems to be an optimal tool that might have a positive impact on the engagement of the patients. In fact, the participants of both experimental conditions showed a high score in satisfaction and the level of engagement in the study.

Another strength of the results of this study is their potential value in the fields of public health and clinical practice due to the positive effects of the two psychological intervention programs delivered through online messages. Mindfulness practice and positive strengthening are current psychological interventions that have recently been shown to be effective for the enhancement of psychological wellbeing and positive affect in patients with CVDs [32,36]. The results of the present study provide further evidence of the suitability of these interventions for the promotion of patients’ wellness, even in a brief format combining mHealth technology. Moreover, according to the results obtained, both psychological interventions were effective in improving self-efficacy beliefs related to disease management as well. Given that research suggests a relationship between positive psychological wellbeing and CVH in healthy [58] and diagnosed individuals [59], the implementation of the psychological intervention programs tested in this study could facilitate the prevention of and, in case of disease development, the recovery from, the ability to cope with, and the adaptation to a new health condition.

Furthermore, these effective mHealth psychological interventions are supported by a theory of behavior change, namely the social cognitive approach of Bandura [12]. Specifically, the fourth source to generate self-efficacy related to physiological states was explored, which leads to better emotional management and improved physiological states. Given that the fourth source of this theory is the least studied, we therefore believed that it had the potential to be the most valuable to test.

### 4.2. Limitations and Future Research

This study has several limitations that should be noted. First, although applying mHealth for brief psychological treatments to patients with CVDs seems feasible, it is also important to note that older adults may have difficulties in using health technologies or technologies overall such as WhatsApp. Therefore, mHealth might not be applicable to all CVD patients. A second limitation is the use of only self-reported measures, without any social or physiological additional measures to compare the results. Future studies combining self-reported measures with more objectives measures are therefore needed to test the stability of the results and potentially extend their implications. Third, given the limited sample size of the study and the fact that the allocation of participants to the groups was not completely randomized, the robustness of the results could have been reduced. In addition, the normality of the data was assumed because the sample size of both experimental groups was greater than 30 and that of the control group was 29; therefore, the use of parametric tests might seem inappropriate. However, considering that the final sample size of the study falls within the sample size effect estimate and the fact that the use of parametric tests in clinical practice does not appear to significantly bias the treatment effect [60], it would be expected that the null hypotheses were correctly rejected. Moreover, the sample size was comparable to other studies with patients with CVDs and eHealth interventions with a somewhat similar design [13,61,62]. Nonetheless, given that this was a feasibility study, future research with a larger number of participants and with full randomization to the experimental and control conditions should replicate the proposed design to test if further support can be provided to the results obtained. Given that rehabilitation interventions with social support have been found to be very helpful for cardiac patients [63] and could be carried out in a group format, we hypothesize that the efficacy of interventions developed in any future study would be stronger in a group format than in an individual format.

Fourth, the fact that 11.43% of the participants dropped out during follow-up measurements may prevent definitive results on the effect of treatments. Although complete-case analysis is suitable for feasibility studies [64], this approach cannot consider the potential effect on missing participants. However, the dropout rate of this study is significantly lower than the mean rate reported in a recent systematic review and meta-analysis of dropout rates in digital interventions in patients with chronic diseases, including CVDs [65], where the overall dropout rate was 43% for observational studies and randomized control trials. Despite this evidence, future studies with a more robust research design, aimed at replicating the current findings, should likely include a method to adjust for any potential bias due to missing data.

In addition, a longitudinal study lasting several years could help to evaluate the influence of other variables over time, such as the influence of economic and educational level. This is especially important considering that the majority of the sample in this study reported a low socioeconomic status and educational level. These characteristics could be due to the fact that most of the participants were retired, which often results in receiving a low pension, and to the fact that formal education was less common in previous decades. However, these characteristics should be taken into account in further studies, as low socioeconomic status may be related to poorer health outcomes. In this regard, a recent study found that patients with CVDs who reported lower income were included in a cluster of low self-reported mental and physical health, which in turn was associated with a profile of poorer psychological wellbeing [49]. Therefore, patients with lower socioeconomic status may benefit even more from these psychological interventions. However, futures studies should include a larger sample with a more diverse economic and educational background to compare the efficacy of these interventions among the individuals from different groups.

Finally, considering the fact that the face-to-face session provided information about the benefits of the mHealth-based intervention, it cannot be ruled out that the effects observed might be due, at least in part, to expectancy or placebo effects, or even to social desirability. Furthermore, given that the two psychological interventions tested in the present research (mindfulness and positive strengthening) have shown effective results in cardiac patient self-evaluation, we believe that a possible combination of both interventions for future studies with cardiac patients could offer promising results. In fact, in the existing scientific literature, such an integrative intervention has been proposed in a non-clinical population, known as the positive mindfulness program [66].

Continuing with future research, other measures of self-reporting could be investigated to evaluate variables such as life satisfaction [67] and quality of life [68]. Thus, other studies could evaluate the evolution of anxiety and depression symptoms over time to determine the impact of long-term intervention on these symptoms. In addition, we also propose the use of physiological measures (e.g., level of cortisol in saliva or heart rate variability) in order to provide objective results that aim to consolidate the results presented in this study. In addition, through mHealth-based interventions, it may be possible to promote self-management behaviors that can help patients to cope with the new circumstances post-COVID-19. The results of this study seem to indicate that the combination of brief psychological interventions, due to their low cost and promptness, together with the adaptation of the treatments to both the rising technological reality and the social isolation as consequence of COVID-19, is an attractive and effective alternative to consider when working with these patients.

## 5. Conclusions

Our study has demonstrated the effectiveness of mindfulness and positive strengthening programs in improving subjective emotional wellbeing (increasing positive affect and reducing negative affect) and management self-efficacy for chronic disease, which, in turn, could improve the physiological state of cardiac patients compared to the TAU group. An innovative intervention format administered through mHealth based on WhatsApp messages was used. This format has proved to be effective by overcoming the limitation of a lack of adherence to face-to-face intervention due to displacement and difficulties in reconciling personal life and treatment. For future studies, this would be an optimal way to reach a large number of cardiac patients and patients with other chronic diseases. Furthermore, a fusion of both interventions, through a positive mindfulness program based on mobile messages could give promising results in cardiac patients.

## Figures and Tables

**Table 1:** Demographic and clinical characteristics.

	Total (*n* = 93)	Mindfulness Group (*n* = 32)	Positive Strengthening Group (*n* = 32)	TAU Group (*n* = 29)	Statistical Significance
Age (*M, SD*)	63.9 (10.9)	64.8 (10.1)	61 (10.3)	66.2 (12.1)	(*F*(2,90)= 1.87, *ns*)
Gender, *n (*%)					(*X*^2^ = 4.59, gl 2, *ns*)
Male	76 (82%)	28	28	20	
Female	17 (18%)	4	4	9	
Marital status *n (*%)					(*X*^2^ = 17, df 10, *ns*)
Single	6 (7%)	4	1	1	
Single with couple	2 (2%)	2	0	0	
Cohabiting partner	1 (1%)	1	0	0	
Married	79(85%)	24	31	24	
Separated	2 (2%)	1	0	1	
Widowed	3 (3%)	0	0	3	
Employment status *n (*%)					(*X*^2^ = 5.4, gl 8, *ns*)
Retired	60 (65%)	21	17	22	
Part-time work	1 (1%)	0	1	0	
Full-time work	22(24%)	7	10	5	
Unemployed	7 (7%)	3	3	1	
Home care	3 (3%)	1	1	1	
Educational level *n (*%)					(*X*^2^ = 19.28, gl 8, *ns*)
Elementary school	68 (73%)	22	23	23	
Middle school	6 (7%)	0	5	1	
High school	11 (12%)	6	3	2	
University	8 (9%)	4	1	3	
Economic level					(*X*^2^ = 14.1, gl 6, *ns*)
<22,000 €	78 (84%)	25	26	27	
22,000–43,000 €	13 (14%)	6	5	2	
>43,000 €	2 (2%)	1	1	0	
Age onset CVD (*M, SD*)	60 (11.5)	61.1 (10.8)	54.1 (13.24)	63.0 (11.8)	(*F*(2,90) = 4.63, *p* < 0.01)
Time with CVD (*M, SD*)	3.82 (6.04)	3.81 (5.10)	5.41 (7.77)	2.07 (4.27)	(*F*(2,39) = 0.097, *ns*)
Type of CVD *n (*%)					
Angina pectoris	31 (33%)	12	13	6	(*X*^2^= 3.1, gl 2, *ns*)
Myocardial infarction	42 (45%)	11	16	15	(*X*^2^= 2.31, gl 2, *ns*)
Hearth failure	8 (9%)	3	1	4	(*X*^2^= 2.24, gl 2, *ns*)
Arrhythmia	9 (10%)	3	3	3	(*X*^2^= 0.02, gl 2, *ns*)
Other	19 (20%)	9	6	4	(*X*^2^= 2, gl 2, *ns*)
More than one CVD *n (*%)	16 (17.2%)	6	7	3	
Level of limitation of ADL *n (*%)					
Level 1	43	19	14	10	(*X*^2^= 3.9, gl 2, *ns*)
Level 2	27	8	9	10	(*X*^2^ = 0.68, gl 8, *ns*)
Level 3	19	3	7	9	(*X*^2^ = 4.45, gl 2, *ns*)
Level 4	4	2	2	0	(*X*^2^ = 1.89, gl 2, *ns*)

Note: TAU—treatment as usual, *SD*—standard deviation, CVD—cardiovascular disease, and ADL—activity of daily life.

**Table 2:** Within-subject effects of the ANCOVA.

Variable	Mean Square	*F*	*df*	Error *df*	*p*	η_p_^2^	Power
Positive affect							
Time	6.22	8.39	2.85	250.78	<0.001	0.09	0.99
Time × anxiety	0.38	0.52	2.85	250.78	0.661	0.01	0.15
Time × depression	1.26	1.70	2.85	250.78	0.171	0.02	0.43
Time × condition	8.56	5.77	5.70	250.78	<0.001	0.12	1.00
Error	65.29						
Negative affect							
Time	4.25	10.83	2.69	236.48	<0.001	0.11	1.00
Time × anxiety	5.13	13.08	2.69	236.48	<0.001	0.13	1.00
Time × depression	0.41	1.04	2.69	236.48	0.370	0.01	0.27
Time × condition	4.47	5.69	5.38	236.48	<0.001	0.12	1.00
Error	34.53						
CMSE							
Time	3.73	10.78	2.12	185.39	<0.001	0.11	0.99
Time × anxiety	0.21	0.61	2.12	185.39	0.552	0.01	0.15
Time × depression	1.09	3.16	2.12	185.39	0.042	0.04	0.62
Time × condition	4.58	6.62	4.21	185.39	<0.001	0.13	0.99
Error	30.46						
SEMCD							
Time	20.78	7.18	2.98	262.35	<0.001	0.08	0.98
Time × anxiety	3.70	1.28	2.98	262.35	0.281	0.01	0.34
Time × depression	8.74	3.02	2.98	262.35	0.031	0.03	0.71
Time × condition	14.05	2.43	5.96	262.35	0.027	0.05	0.82
Error	254.55						

Note: CMSE = cardiovascular management self-efficacy; SEMCD = self-efficacy for managing chronic disease.

**Table 3:** Levels of engagement and general satisfaction for both intervention groups.

	Mindfulness *M* (SD)	Positive Strengthening *M* (SD)	ANOVA *F* (1,62); *p*
Post-test 1			
Engagement	4.54 (0.66)	4.33 (0.85)	1.29, 0.26
General satisfaction	9.09 (0.92)	8.73 (1.44)	1.51, 0.22
Post-test 2			
Engagement	3.67 (1.02)	4.03 (0.86)	2.42, 0.13
General satisfaction	8.85 (1.44)	8.97 (1.03)	0.15, 0.70
Post-test 3			
Engagement	3.88 (0.98)	3.75 (0.88)	0.29, 0.59
General satisfaction	9.31 (1.18)	9.00 (1.02)	1.29, 0.26

Post-test 1 (after messages); post-test 2 (after task maintenance); post-test 3 (follow-up).

## Data Availability

The data presented in this study are available on request from the corresponding authors.

## Supplementary Materials

The following supporting information can be downloaded at: https://www.mdpi.com/article/10.3390/jpm12121953/s1, File S1. Annex 1. Mindfulness messages and Annex 2. Positive strengthening messages.
