# Supplementary material for: Effectiveness of Mindfulness and Positive Strengthening mHealth Interventions for the Promotion of Subjective Emotional Wellbeing and Management of Self-Efficacy for Chronic Cardiac Diseases
Source: J Pers Med. 2022 Nov 25;12(12):1953. doi: 10.3390/jpm12121953 (PMC9784634; doi:10.3390/jpm12121953)
Supplement: Supplementary file 1 [file jpm-12-01953-s001.zip › jpm-2041644-Supplementary Materials.docx]

**Annex 1. Mindfulness messages**

**Welcome message**

Dear participant, the messages you will receive during the next two weeks will not take you much time out of your daily routine, just 10 minutes that you will manage at the time of the day that best suits you. The important thing is that you carry out the activity that we propose each day, although reserving a time at the same time each day facilitates the habit. Thank you for your participation!

**Message 1. Good morning!**

We ask you to stop for 10 minutes. Adopt a comfortable posture, in which you can keep your back straight and relaxed at the same time. Now, focus all your attention on your breathing. Take a breath and let go, without forcing anything. No need to change your breathing rhythm, just observe it. Inspire and exhale, inhale and exhale... Repeat during these minutes. This is your moment.

**Message 2. Good morning!**

For this exercise, you can close your eyes. We ask you to focus on your body and how you perceive it. You can make a mental scan of your entire body, stopping a few moments for each part. You may notice some discomfort, some pain, etc., or pleasant sensations. You do not have to do anything else, just perceive body sensations, accepting them as they are and noticing how you feel them.

**Message 3. Good morning!**

Take 10 minutes to contemplate the space that surrounds you in detail. Pose your gaze on each object, observe it with full attention, such as if it were the first time you see it. Discover everything that is around you at this time. You can look at the color, the shape of each object, observing its characteristics. The idea is to focus your attention on what you observe. Remember that this is your moment.

**Message 4. Good morning!**

For this exercise, you can sit in a park or garden, walk in a quiet place or stop a few minutes in the place where you are. We ask you to close your eyes if you are sitting and perceive in detail the sounds that surround you. Take a few minutes to pay attention to the sounds you perceive and the sensations that they generate to you.

**Message 5. Good morning!**

For this activity, close your eyes and touch the surfaces of the objects that are around you. Feel the texture different from each object, smooth, rough, soft, etc. In addition, you can caress your skin; feel your own skin, all with full attention. This is your moment to feel through touch.

**Message 6. Good morning!**

It is your moment. Take a few minutes to stop and smell several pleasant fragrances. For example, the smell of flowers in a garden, the delicious smell of fresh coffee or a perfume that pleases you, etc. Center all your attention in experiencing that smell and in the sensations that produce it. At the same time, you can close your eyes if you find it comfortable to focus your attention more on detecting the smell.

**Message 7. Good morning!**

Delight yourself with a flavor you are passionate about. For example, prepare an infusion that is enjoyable (or any other beverage you like). Your mission is to center all your attention on the taste, in each small and serene sip. You can pay attention to the food you take at breakfast, lunch or dinner, savoring the food, distinguishing the flavors. If possible, make sure there is no noise (e.g., TV) to appreciate better the flavors during that time.

**Message 8. Good morning!**

It is the moment to pay full attention when eating. To unite all that has been practiced until now, we ask you to pay full attention again during the meal. In this time trying to experiment with all your senses at once: observing the food and other objects that are nearby, realizing the pleasant aromas, the flavor of each food, as well as the texture of the food it touches, such as bread, or the objects it is using.

**Message 9. Good morning!**

It is time to recognize your emotions! How are you feeling in this precise moment? Stop a few minutes and observe the feelings you are experiencing, whether they are pleasant, unpleasant or neutral. Whatever they are, they are fine. Just uncover them and observe them during the next minutes.

**Message 10. Good morning!**

Today we ask you to pay attention to your thoughts, to the content of them. Possibly, your thoughts are varied and jump from the past to the present and future. They may be positive, negative or neutral. Do not try to change them. Simply observe them.

**Message 11. Good morning!**

Today we ask you to do a little relaxation exercise. Your goal is to discover the thoughts and feelings that are presenting during the time you choose to perform the exercise. How they cross each other and as a memory or image causes a certain feeling, and vice versa. Do not let yourself be trapping by them, just watch them.

**Message 12. Good morning!**

Inspire, exhale… Take a breath and then release it. Connect with your breath. No place to go or anything to do. Remain simply by observing everything that happens while you breathe, both around you and in your interior, with a kind and compassionate attitude towards yourself.

**Message 13. Good morning!**

With what you have learned so far, you can apply the full attention in your day to day. Any time of your day is adequate. You can stop for a few minutes and focus on your breathing, your bodily sensations or in the sense of sight, hearing, touch, smell, taste... We encourage you to take a few minutes to experience these sensations. The right time is always now.

**Message 14. Good morning!**

Thank you for participating in this study! This is your last message. Now think of a simple plan to be focused in the present, which you can commit to do during the next few days. We ask you to reserve 10 minutes each day to carry out any of the recommendations you have made these days. For example, you can connect with yourself through breathing, or by paying attention to your environment, etc. Reserve a specific moment of the day and make it for two weeks.

**Annex 2. Positive strengthening messages**

**Welcome message**

Dear participant, the messages you will receive during the next two weeks will not take you much time out of your daily routine, just 10 minutes that you will manage at the time of day that best suits you. The important thing is that you carry out the activity that we propose each day, although reserving a time at the same time each day facilitates the habit. Thank you for your participation!

**Message 1. Good morning!**

Time for a positive exercise! Stop for a few minutes and think or write three good things that happened yesterday.

**Message 2. Good morning!**

It is time to count gestures of kindness! How? During this day, keep track of those kind acts you perform, as well as those you notice in other people. At the end of the day, remember the account of all the generous and kind acts that have taken place.

**Message 3. Good morning!**

Time for a positive exercise! Think in detail about a successful event in your current life (small or large) and how it contributed to that success.

**Message 4. Good morning!**

A door closes, another door opens! Consider a moment in your life when a negative event led to positive consequences that you did not expect. Tell it as a life experience to someone close to you.

**Message 5. Good morning!**

Choose three fun things that have happened in your day. Why did they happen? Was it you who caused them directly, was it spontaneous or was it someone else? The ability to laugh at yourself and adversity is a very positive thing; it helps you not to take things too seriously.

**Message 6. Good morning!**

Time for a positive exercise! Take time to stop and appreciate something positive around you today: a good friend, a beautiful sight or the kind act of another person.

**Message 7. Good morning!**

Today has a positive mission! Take the opportunity to do a kind act (big or small) for someone during today, and then notice how you feel when you do it.

**Message 8. Good morning!**

Take a few minutes to express your gratitude (by phone or in person) to someone who has done something good for you (today or years ago).

**Message 9. Good morning!**

It is time to recognize your achievements! Think of an event that you have lived in which you have been able to give your best. Allow yourself to experience that pride towards yourself. Enjoy it!

**Message 10. Good morning!**

Today has a positive mission! When today someone tells you positive news, investigate and prolong your interest in that person, through active listening, dedicating your time.

**Message 11. Good morning!**

Close your eyes and visualize the face of someone who is still alive and who years ago did or said something that changed your life for the better. Someone whom you never thanked properly and can thank for the next few days.

**Message 12. Good morning!**

Close your eyes and visualize your future thinking of something you would like to achieve. Focus on the differences with the present moment and on how you will get those changes or those goals you hope to achieve.

**Message 13. Good morning!**

It is time to cultivate a positive outlook! Try to modify your point of view when you are being assaulted with repetitive negative thoughts, looking for something positive or pleasant around you.

**Message 14. Good morning!**

Thank you for participating in our study! This is your last message. Think of a simple plan to be more positive to which you can commit during the next few days (for this we recommend writing in one diary three positive things that have happened each day before going to sleep). Start now and follow it for the ne
